# Effects of Resident Education and Self-Implementation of Integrated Pest Management Strategy for Eliminating Bed Bug Infestation in Ahvaz City, Southwestern Iran

**DOI:** 10.18502/jad.v14i1.2705

**Published:** 2020-03-31

**Authors:** Ismaeil Alizadeh, Elham Jahanifard, Mona Sharififard, Mohammad Ebrahim Azemi

**Affiliations:** 1 Infectious and Tropical Diseases Research Center, Health Research Institute, Ahvaz Jundishapur University of Medical Sciences, Ahvaz, Iran; 2 Department of Biology and Vector Control, School of Public Health, Kerman University of Medical Sciences, Kerman, Iran; 3 Department of Medical Entomology and Vector Control, School of Public Health, Ahvaz Jundishapur University of Medical Sciences, Ahvaz, Iran; 4 Department of Pharmacognosy, Medicinal Plant and Natural Product, Research Center, School of Pharmacy, Jundishapur University of Medical Sciences, Ahvaz, Iran

**Keywords:** Bed bug, Urban pest, Integrated pest management, Strategy

## Abstract

**Background:**

Bed bugs as blood-sucking insects have become a public health problem in urban communities throughout the world. The objective of this study was to determine the effects of resident education and self-implementation of Integrated Pest Management (IPM) strategy for eliminating bed bug infestation in infected apartments in Ahvaz City, Iran.

**Methods:**

This interventional study was conducted in seventy apartments infested with bed bug (*Cimex lectularius*) in Ahvaz City, southwest Iran, during a 6-month period. The bed bug infestations reported to Health centers were inspected visually and confirmed by medical entomology experts. Then, the heads of the households were received techniques/tools of the IPM program and trained by medical entomology experts before self-implementation of control methods. Finally, the infected apartments were inspected by the experts at 1, 3, and 6 months after intervention and data were recorded in a checklist.

**Results:**

From the seventy infected apartments, 57%, 28% and 15% were considered as low, moderate, and high level infestation respectively. The bed bug infestation was eliminated from 53 apartments (76%) after one month and it reached to 62 apartments (88%) by the end of third month. Finally, after six months of applying IPM program, bed bugs infestation was eliminated from all infected apartments (100%). Residents expressed their 100% satisfaction with applying the bed bug IPM strategy.

**Conclusion:**

Training residents to implement the IPM program can reduce pest control costs, the volume of pesticides consumed, and human exposure to chemical pesticides, resulting in increased human and environmental health and safety.

## Introduction

The bed bugs (*Cimex lectularius* and *C. hemipterus*) as blood-sucking insects have become a serious pest and public health problem in urban communities throughout the world. They affected human health through blood feeding that can lead to pain, itching, secondary infections, loss of sleep, psychological distress and other allergic responses to humans (1–8).

These urban pests have adapted to live in close to indoor human environments including houses, hotels, dormitories, sports, workplace environments, and transport systems all over the world during the past decade (1, 2, 5, 9). Generally, increased international travels, insecticide resistance, climate changes, lack of awareness, lack of effective monitoring and management tools and the decreased use of broad-spectrum insecticides in human dwellings can be outlined as leading reasons involve with the bed bug emerging (2, 5, 9–11). Also social interactions between residents, home visits, or exchange of infested furniture are revealed to cause bed bug distribution in recent studies (5, 12, 13).

Elimination of bed bug infestation is costly and time-consuming because of activity and nocturnal blood feeding, habit of hiding, spreading around the house (5, 14, 15). They are providing to be one of the most challenging urban pests. Most people prefer to use chemical insecticides for removing the bed bug infestation due to more convenient application of chemical method compared with other control tools. Even if relatively proper insecticides are used, the challenges with pesticide resistance can occur. Also, using chemical insecticides directly on furnishings in human dwellings increases the risk of human exposing to pesticides (1, 5).

Integrated pest management (IPM) approach is considered as a promising, comprehensive and safe strategy for humans and environment recently. It exploits cooperation between residents and professional pest experts to use both chemical and non-chemical tools and technics simultaneously to eliminate of pest infestation (13). Non-chemical techniques include heat treatment, vacuuming, mattress encasements, laundering, freezing and heat steam (3, 5, 14, 16, 17). In order to effectively eliminate bed bug infestations, recognition, management, and eradication are the components of successful control program (13).

Bed bug infestation is increasing in Ahvaz City, south west of Iran based on health services reports, as well as resident’s reports to Department of Medical Entomology. The average cost of bed bug control is about $ 0.78 per m^2^ for chemical control based on pest control services reports in Ahvaz City in 2019. While the bed bug infestation usually occurs in low-income communities, control by those companies is not cost-effective and the people prefer to apply some conventional control methods by themselves.

Little studies have been documented the usefulness of IPM strategy for control bed bugs (5, 13, 18, 19). But there are no studies on resident education and self-implementation of IPM strategy for control of bed bug infestation. The objective of this study was to determine the effects of resident training to apply IPM strategy for eliminating bed bug infestation in infected apartments.

## Materials and Methods

### Design of Interventional study

**Fig. 1.**
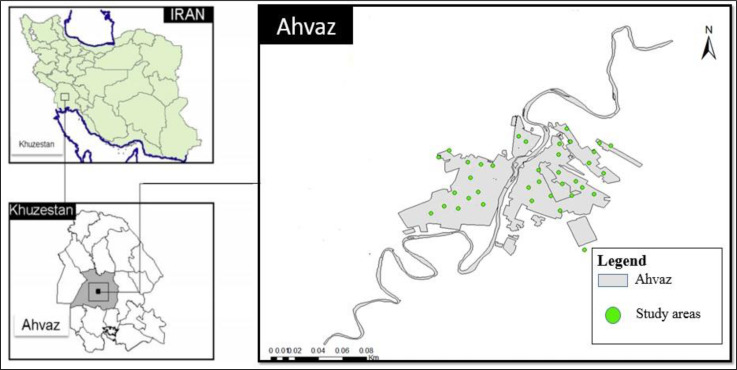
Study areas in Ahvaz City, southwestern Iran

This interventional study was one arm pre-post study conducted in 70 apartments with bed bug infestation in Ahvaz City, southwest Iran during a 6-month period from May 2015 to October 2016. Ahvaz City is one of the metropolitan cities in the southwestern of Iran, with a population of 1.3 million. It is located in a dry area of Iran with subtropical hot desert climate. The infected apartments were located in different parts of the city both in west and east of the Karun River (Fig. 1). All the apartments were including bedroom, living room, kitchen, and bathroom.

At first, a list of the apartments with reported bed bug infestation was prepared from the health centers and Department of Medical Entomology of Ahvaz Jundishapur University of Medical Sciences in Ahvaz City.

**Fig. 2.**
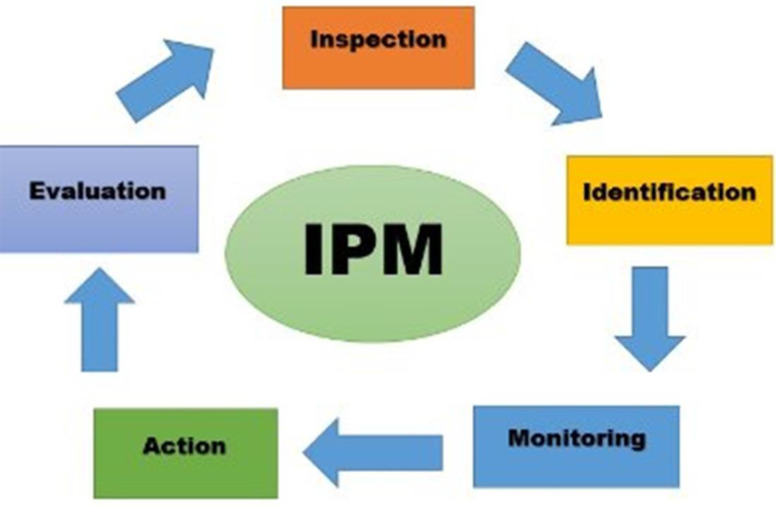
Different steps of running an Integrated Pest Management program

**Fig. 3.**
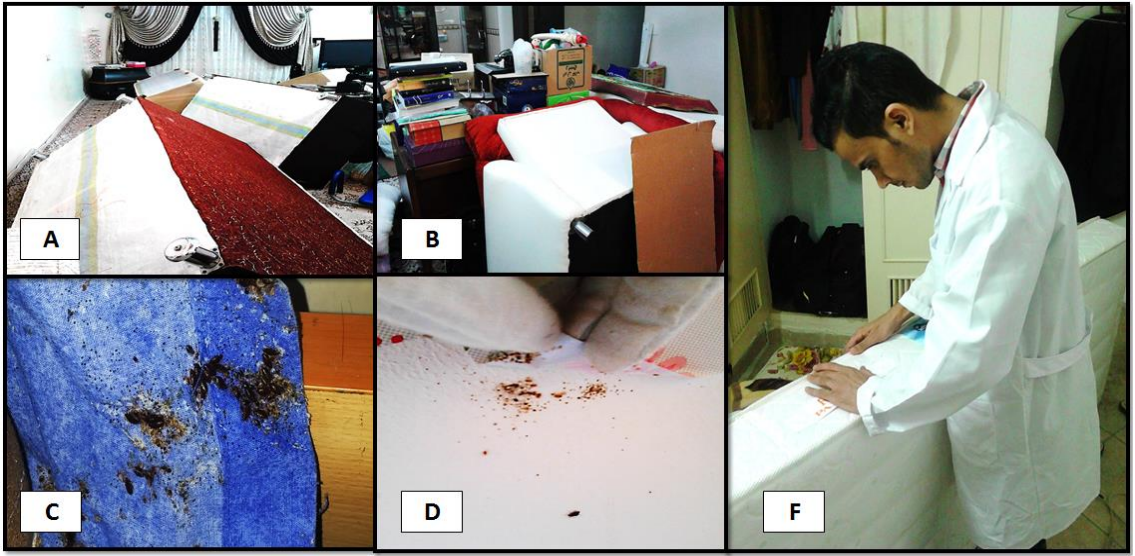
(A) and (B) Infested living room, (C) Common Bed bug colony over the bed, (D) Bed bug infestation under wallpapers (F) Visual inspection by medical entomology expert

Inspection of the infected site is the first step of each IPM program (Fig. 2). The houses were inspected visually by medical entomology experts to confirm the bed bug infestation (Fig. 3). From the reported infected houses, 70 houses were selected randomly for the study. Residents of infected apartments were informed about the objectives and procedures of the investigation. All the heads of households signed written consent prior to implementation of the program.

A self-administered checklist was used to evaluate the bed bugs infestations level. The bed bug infestation was assessed through the visual observation of live and dead bed bugs, fecal spots, cast skins, and eggs and categorized to three levels of low, medium and high infestation (Low: 1–10 live or dead bed bugs, few fecal spots and few cast skins; medium: 11–50 live and dead bed bugs, fecal spots and cast skins, high: > 50 live or dead bed bugs, lots of fecal spots and cast skins). Bed bugs were collected and preserved in 75% ethanol and sent to the laboratory of Medical Entomology and Vector Control for identification.

### Training of residents

**Fig. 4.**
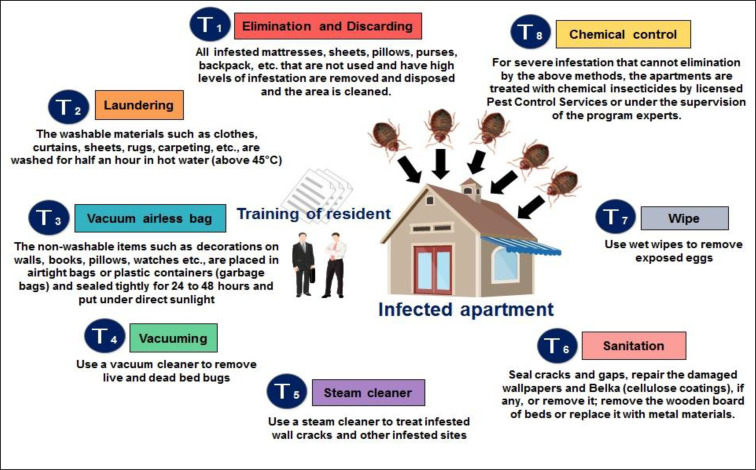
Techniques/tools of the integrated bed bug control program

**Fig. 5.**
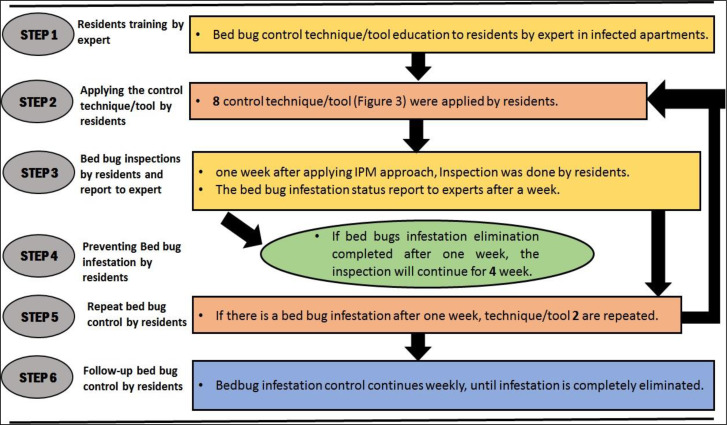
Flowchart of applying the IPM Program in bed bug infested houses in Ahvaz City, southwest Iran

After inspection, identification of the infestation, signing the consent form, the head of the households were trained by medical entomology experts to control the bed bug infestation by self-applying of IPM strategy. They also provided guidelines to improve the prevention and control programs based on IPM approach. Eight techniques/tools and six steps were considered in the bed bug management program are explained in (Figs. 4 and 5). Totally two hours was spent to train the head of each house in this survey. The steps of T1, T3, T7 and T8 were performed only once by residents in the first week but the use of other steps includes laundering, vacuuming, steam cleaner and wipes were continued several times until bed bug elimination (Fig. 4). Entomological experts were on-call with the residents until complete bed bug elimination.

### Data Collection

The infected apartments were inspected by the entomological experts at 1, 3, and 6 months after intervention. The bed bug infestations in each home were recorded in self-administered checklist based on visual inspection. Also, after one, three and six months, the residents were interviewed for three questions: (1) Do you still have bed bug infestation in your apartment? (2) How effective do you think this program is? (3) How worried you are about bed bug infestation? A total of 241 residents from seventy infected apartments were interviewed.

### Data Analysis

The data were collected to determine the overall effectiveness of IPM strategy in infected apartments. Excel Version 2013 software was used to analysis the frequency and descriptive variables.

## Results

### Evaluation of the self-implementation Integrated Pest Management program

Totally, 70 infected apartments were inspected during this survey. The levels of infestation were recognized as low, moderate and high in 57%, 29% and 15% of infected houses based on initial inspection. The collected specimens were identified as common bed bug (*C. lectularius*). During the home inspection phase, it was found that the infestations rate of bedrooms and living rooms were 73% and 27%, respectively.

**Fig. 6.**
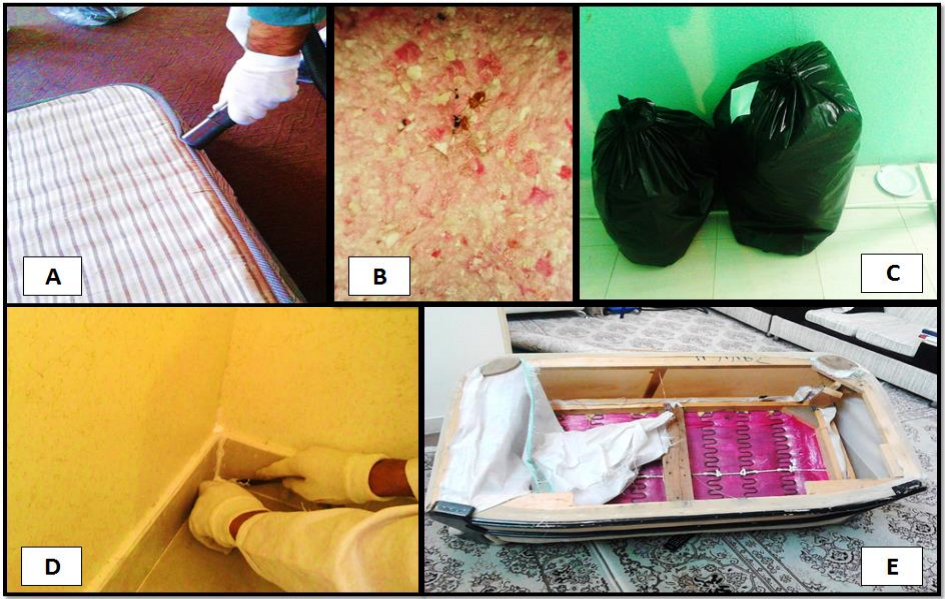
(A) Vacuuming, (B) Infested Belka in living room, (C) Vacuum airless bag, (D) Sealing wall cracks and crevices, (E) Discarded wooden furniture, photo by Ismaeil Alizadeh

**Fig. 8.**
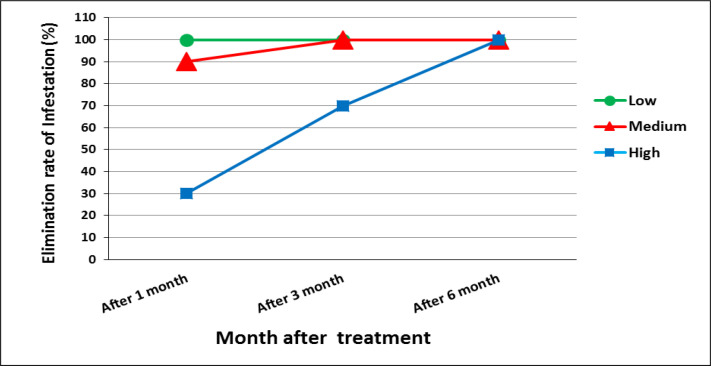
Effect of Integrated Pest Managaemnt (IPM) on bed bug eliminating rate with low, moderate and high level of infestation

Also in 38%, 32%, 18% and 14% of the infected houses, the bed bug harborages were crack and cervices, bedding, wooden furniture and wallpaper and Belka, respectively (Fig. 8). Residents of all apartments attended to the integrated bed bug control program in this study (Fig. 5). The bed bugs infestations from 53 apartments (76%) were eliminated after one month. In this time 100%, 90% and 30% of apartments with low, moderate and high level of infestations were cleaned from bed bug infestations, respectively. The bed bug infestation was eliminated from apartments with low infestations without using pesticide. Then, the bed bug elimination from 62 apartments (88%) was occurred after three months of self-implementation of IPM strategy. In this time 100% and 70% of apartments with moderate and high levels of infestation were cleaned, respectively. Finally, after six months of applying IPM strategy by residents, the bed bug infestation was eliminated from all infected apartments (100%) (Fig. 6). All residents have high cooperation in this study. In total, all residents expressed their 100% satisfaction with applying the IPM strategy. As shown in (Fig. 6), elimination of bed bugs from apartments with low and moderate level of infestation occurred faster than those with high bed bugs infestations.

**Fig. 7.**
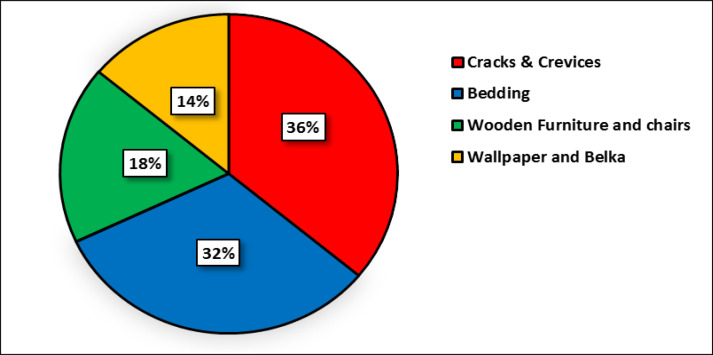
Bed bugs infestations rate in different sources of the infected apartments

### Resident’s interview

During this survey, 286 people were lived in the infested apartments. From them, 241 people with the age of > 16 years were interviewed. All of them were satisfied with IPM strategy as an effective approach for bed bug elimination. Bed bug bite signs were observed in 23% of the residents. Among the interviewed resident, 82% were very concerned, experienced sleep loss, and psychological distress, 10% of them had a somewhat concern and 8% showed no concern.

## Discussion

The role of education in raising knowledge of the public and consequently reducing the prevalence rate of vectors and vector-borne diseases is completely clear. A study by Alizadeh et al. (2018) in Ahvaz City reported residents’ knowledge about transmission of bed bug, medical importance, habitat, infestation control, prevention of bed bug were 36.7%, 13.3%, 35%, 75% and 66.7% respectively (20). Most people get their necessary information by searching the internet, but they are not practically familiar with pest control techniques. Our study is the first documented practically application of self-implementation IPM strategy in order to eliminating bed bug infestation in Iran. The purpose of this study was to examine the overall effectiveness of IPM approach to removing bed bug infestation, not to evaluate specific techniques/tools. The IPM program was attributed to five practices including: A) Inspection of infected apartments, B) Identification of insect species, (C) Monitoring the infestation level, D) Action including training residents to apply the IPM techniques/tools according to protocol, (E) Evaluation the effectiveness of control program.

Education of residents regarding biology, behavior, medical importance of bed bug, and what actions should be to take for infestation removing is an important component of any IPM effort in multifamily housing (13).

Bed bug infestation was eliminated completely (100%) using non-chemical techniques/tools in houses with low infestation in our study. This finding is confirmed by Wang et al. 2016, Cooper et al. 2.16. They also reported that residents training, non-chemical techniques/tools, and periodic monitoring of bed bug can effectively remove light bed bug infestations (5, 13). Although the IPM strategy is time consuming, reducing the use of pesticides and exposing them to humans are two of the main goals of an IPM program that can offset the time delay.

Generally, integrated pest management strategy which uses various non-chemical techniques/tools before chemicals becomes more prevalence and promising in last decades. Furthermore, IPM considered as practical strategy in urban environment where bed bug control is challenging in low-income communities and multiunit housing building (18). Due to the high cost of bed bug elimination (about $ 0.78 per m^2^) by pest control services, residents in low-income communities prefer to control the infestation themselves. Therefore, the role of education of residents and their involvement in the implementation of the pest control program can reduce their costs (1). A few studies have documented the effectiveness of IPM strategy for reducing or eliminating of bed bug infestation around the world (1, 5, 9, 13, 19).

Ongoing education and commitment of the housing residents will play an important role in program success and elimination of bed bug infestation (13). We continued our inspection and training weekly until complete clean-up of the houses. These actions resulted in complete removing of bed bug infestation after one, three and six months in 76%, 88% and 100% of the houses, respectively. In the study of Cooper et al. (2015), bed bug infestation was eliminated from 52 apartments out of 62 treated apartments using IPM strategy and the mean bed bug counts was reduced to 96% after 6 month of inspection treated apartments. Moreover, the infestation rate was decreased from 15 to 2.2% after 12 months (13).

The effectiveness of IPM strategy for deleting bed bug infestation was reported by all the residents. The results of our study are also consistent with Wang et al. (2019) which reported IPM program as a much more effective approach for building-wide control of bed bugs than conventional pest control service(19).

Generally, the failure of IPM program and the prolonged infestation are probably related to not completely follow-up the control instruction, lack of resident cooperation (5, 10, 13, 21) or bed bug re-infestation of the houses. Additionally, physical disabilities, financial problems, no concern about bed bug infestation and resident‘s distrust to non-chemical methods can be the reasons of lack cooperation of residents (9). Continuous monitoring and evaluation of control operations are two key factors in assessing the effectiveness of every IPM program (19).

Close supervision of medical entomology experts until the full bed bug elimination have been the strengths of this study in addition to high level of resident’s cooperation. Residents’ home attendance during weekly monitoring was one of the drawbacks of the program, making it difficult to accurately estimate the infestation level and quality of the program execution. Also, the reduction in bed bug infestation during the implementation of the program was not quantitatively estimated.

## Conclusion

The present study provides first evidence for using IPM strategy in control bed bugs infestation in Iran. This study was focused on training the residents of infected apartments to eliminate the bed bug infestation using IPM strategy. In addition, this present research provided a good model that is effective for self-implementation in infected apartments suffering from bed bug infestations in Ahvaz City. In conclusion, to get rid of the bed bugs infestations, residents must have high motivation and cooperation in this model. The use of sticky trap and dusts (silica gel or diatomaceous earth± pyrethrum and PBO) (17) in combination with the IPM strategy is suggested in future studies. We also recommend that residents who involve in controlling this urban pest can be trained by mobile health application (22) to improve knowledge and awareness of IPM strategy and arrive to successful bed bug control.
